# Bullous pemphigoid associated with dipeptidyl peptidase‐4 inhibitor showing unfavorable outcomes despite immediate discontinuation of medication

**DOI:** 10.1002/ccr3.3047

**Published:** 2020-08-30

**Authors:** Yasutake Shinohara, Toshie Iijima, Shintaro Sakurai, Teruo Jojima, Eriko Ohira, Shujiro Hayashi, Isao Usui, Ken Igawa, Yoshimasa Aso

**Affiliations:** ^1^ Department of Endocrinology and Metabolism Dokkyo Medical University Tochigi Japan; ^2^ Department of Dermatology Dokkyo Medical University Tochigi Japan

**Keywords:** bullous pemphigoid, dipeptidyl peptidase‐4 inhibitor, type 2 diabetes

## Abstract

We experienced two cases of dipeptidyl peptidase‐4 (DPP‐4) inhibitor‐associated bullous pemphigoid (BP) showing an unfavorable course despite its discontinuation. Clinicians should carefully monitor the course of DPP‐4 inhibitor‐associated BP even after withdrawal of DPP‐4 inhibitor therapy, especially in very elderly patients.

## Graphical abstract

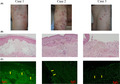

## 1 INTRODUCTION

We report three cases of dipeptidyl peptidase‐4 (DPP‐4) inhibitor‐associated bullous pemphigoid (BP), two cases of which had an unfavorable course despite its discontinuation. Therefore, clinicians should pay close attention to the clinical course of DPP‐4 inhibitor‐associated BP, even after withdrawal of these drugs.

Bullous pemphigoid (BP) is a bullous autoimmune skin disease that is characterized by autoantibodies targeting BP180 and BP230, which are two hemidesmosomal proteins localized at the epidermal‐dermal junction.^1^ BP is relatively common among elderly people, mainly affecting the trunk, lower limbs, and face.^1^ Various drugs have been reported to show an association with BP.^2^ DPP‐4 inhibitors are oral antidiabetic drugs that inhibit degradation of incretins (gastric inhibitory peptide and glucagon‐like inhibitory peptide −1). Recently, there have been several reports that use of dipeptidyl peptidase‐4 (DPP‐4) inhibitors is associated with an increased risk of BP, with the highest risk being noted for vildagliptin among these drugs.^3^, ^4^, ^5^, ^6^ A recent report has demonstrated clinical features and pathophysiology of BP associated with DPP‐4 inhibitors in Japanese patients,^7^ suggesting that noninflammatory BP may be associated with DPP‐4 inhibitors. Several studies have shown that the clinical outcome is better if DPP‐4 inhibitor therapy is discontinued when BP is diagnosed.^8^, ^9^, ^10^

However, we experienced two cases of BP associated with DPP‐4 inhibitor therapy showing unfavorable outcomes despite immediate discontinuation of the relevant drugs. Here, we report a total of three cases of DPP‐4 inhibitor‐associated BP in patients with type 2 diabetes, summarizing their characteristics and the clinical course of BP after discontinuation of DPP‐4 inhibitors. Patients provided informed consent for publication of these cases.

## 2 CASE REPORTS

**Figure 1:**
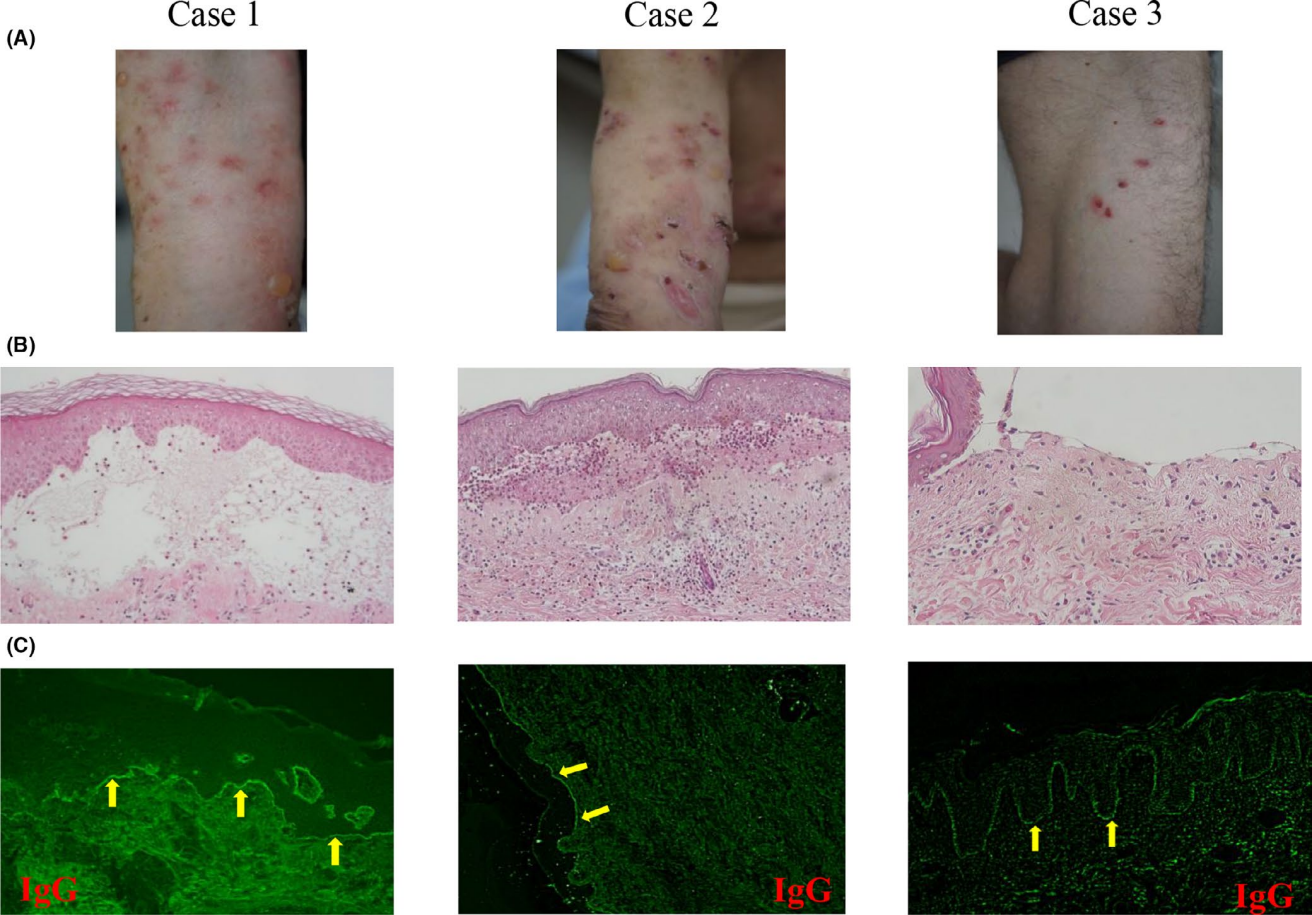
A, Clinical features of skin in Case 1‐3. B, Histopathological findings with hematoxylin and eosin staining of skin biopsy in Case 1‐3. C, Direct immunofluorescence for IgG of skin biopsy in Case 1‐3

Case 1: An 82‐year‐old woman with a long history of type 2 diabetes started to take linagliptin (5 mg/d) in addition to the combination of basal insulin and a glinide. Nine months later, linagliptin was switched to teneligliptin (20 mg/d) at a different hospital. After a further six months, she presented with pruritic erythema on the trunk and limbs, followed by development of blisters on the left arm (Figure 1A). She consulted a dermatologist at our university hospital. Examination revealed diffuse bullae and generalized edematous erythema. Histological examination of a skin biopsy specimen with hematoxylin and eosin (H‐E) staining showed subepidermal blisters and scanty eosinophil infiltration (Figure 1B). Direct immunofluorescence demonstrated linear deposits of immunoglobulin G (IgG) along the epidermal basement membrane (Figure 1C; yellow arrows). The serum level of anti‐BP180 antibody (noncollagen 16A domain; NC16A) was elevated to 328 U/mL. BP was diagnosed from these findings. HbA1c was 7.0% at the diagnosis of DPP‐4 inhibitor‐associated BP.

**Figure 2:**
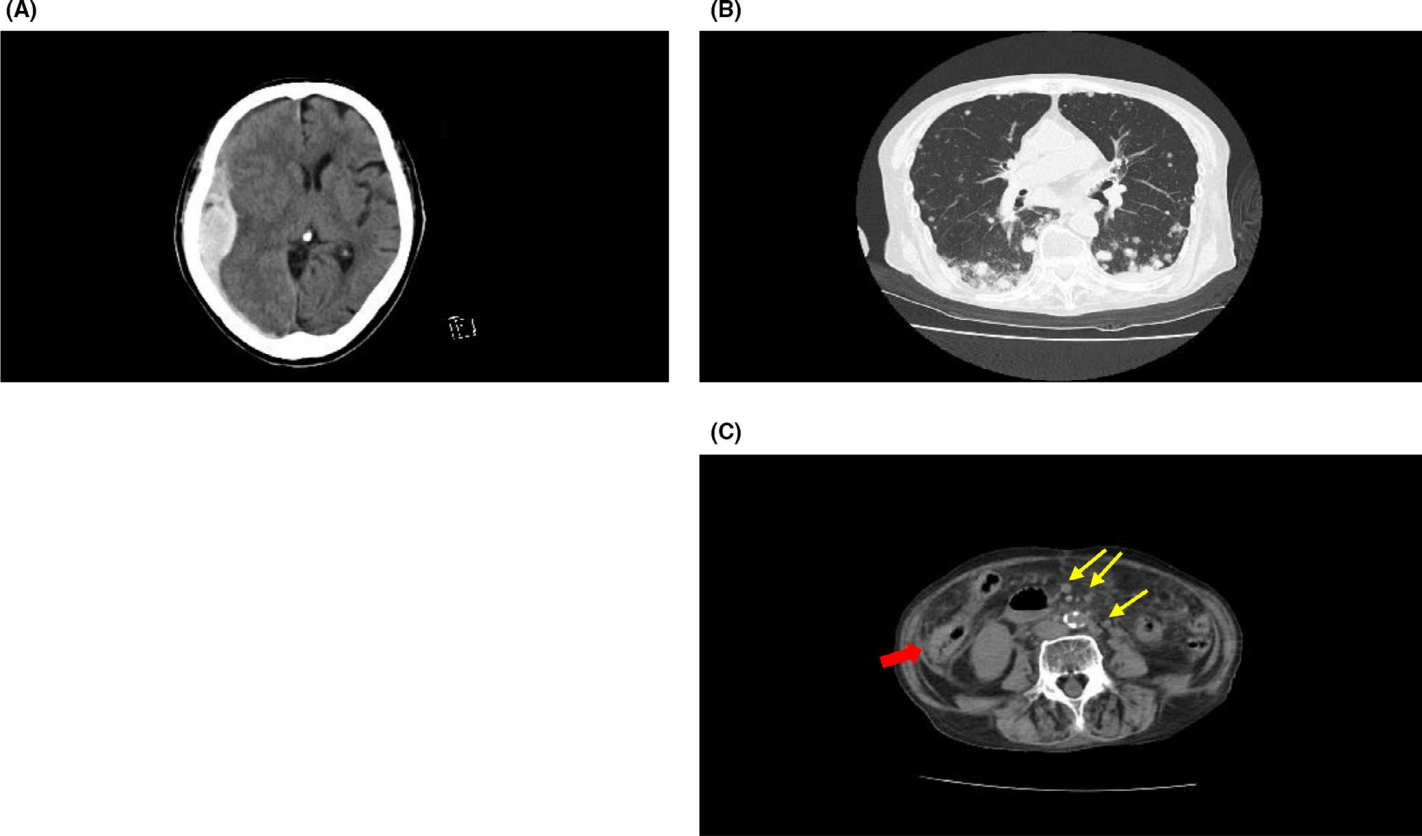
A, Computed tomography of brain in Case 1; chronic subdural hematoma. B, Computed tomography of lungs in Case 2; multiple nodular lesions in the middle to lower lobes of both lung. C, Computed tomography of abdomen in Case 2; suspected ascending colon cancer (red arrow) and para‐aortic lymphadenopathy (yellow arrows)

After hospitalization, treatment with prednisolone (40 mg/d) and cyclosporine (150 mg/d) was initiated, while teneligliptin was discontinued because it was suspected to be the cause of BP. However, her skin lesions did not improve and serum anti‐BP 180 antibody increased to 10 000 U/mL on January 10, 2017. Intravenous steroid pulse therapy was commenced, as well as administration of intravenous immunoglobulin (IVIG) and plasmapheresis on several occasions. Subsequently, her skin lesions improved and anti‐BP180 antibody decreased to 109 U/mL on March 13, 2017. However, her symptoms showed repeated exacerbation after discharge from hospital. Therefore, the patient was readmitted and received intravenous steroid pulse therapy, IVIG, and plasmapheresis, but her skin lesions did not respond. After multiple courses of intravenous steroid pulse therapy followed by a high dose of oral glucocorticoid, HbA1c was increased to 8.4%. After that, she had a fall and developed confusion. Emergency head computed tomography revealed new bleeding into an existing chronic subdural hematoma (Figure 2A). The patient died two weeks later, possibly from cerebral herniation.

Case 2: An 89‐year‐old woman with long‐standing type 2 diabetes started treatment with sitagliptin (50 mg/d), which was switched to vildagliptin (100 mg/d) at a different hospital. Three years after initiation of vildagliptin therapy, she presented with erythema and small tense blisters on her limbs and trunks. She visited a local dermatology clinic and was referred to the department of dermatology at our university hospital. On examination, she had tense bullae and erythema on her chest and limbs (Figure 1A), along with scars due to scratching on her back. Histological examination of a skin biopsy specimen from the right leg with H‐E staining revealed blisters with fibrin precipitates and infiltration of eosinophils into the epidermis and dermis (Figure 1B). Direct immunofluorescence showed linear staining for IgG along the epidermal basement membrane (Figure 1C; yellow arrows). Serum anti‐BP180 NC16A antibody was elevated to 3450 U/mL. BP was diagnosed on the basis of these findings. HbA1c was 7.9% at the diagnosis of DPP‐4 inhibitor‐associated BP. Vildagliptin was discontinued immediately, and treatment was started with prednisolone (30 mg/d), doxycycline (100 mg/d), and niceritrol (500 mg/d). Despite this regimen, new blisters continued to appear, so cyclosporine (150 mg/d) was added to her therapy. Subsequently, the skin lesions gradually improved and did not relapse when prednisolone and cyclosporine were tapered. She continued prednisolone at a dose of 10 mg/d with no recurrence of blistering and erythema. HbA1c was gradually increased and remained at a high level of 8 to 9%. Three months later, she became unconsciousness after a history of poor health for several days. Cardiopulmonary arrest occurred when she was transported to the emergency department of our university hospital. Although emergency treatment was initiated, the patient could not be revived. Laboratory tests performed upon arrival showed leukocytosis of 27 400/µL and elevation of serum C‐reactive protein to 11.91 mg/dL. Whole body computed tomography revealed multiple nodular lesions in the middle to lower lobes of both lung (Figure 2B) and suspected ascending colon cancer (Figure 2C; red arrow) with para‐aortic lymphadenopathy (Figure 2C: yellow arrows).

Case 3: A 67‐year‐old man with type 2 diabetes commenced treatment with alogliptin (12.5 mg/d). The dose of alogliptin was subsequently increased to 25 mg/d and metformin (500 mg/d) was added. After 3.5 years, he developed generalized blisters on his skin and intraorally. He presented to the department of dermatology at our university hospital. On examination, several areas of edematous erythema without blistering were found on his thigh and in the oral cavity (Figure 1A). Histological examination of a skin biopsy specimen with H‐E staining showed subepidermal blisters with scanty eosinophil infiltration into the skin (Figure 1B). Direct immunofluorescence demonstrated linear deposits of IgG along the epidermal basement membrane (Figure 1C; yellow arrows). Serum anti‐BP180 antibody was negative, but BP was diagnosed from the other findings. HbA1c was 7.2% at the diagnosis of DPP‐4 inhibitor‐associated BP. Alogliptin was discontinued immediately and treatment with prednisolone (20 mg/d) was started. The dose of prednisolone was tapered to 4 mg/day as his symptoms gradually improved, and he currently remains in remission. Although HbA1c was increased to 8.6% after treatment with oral glucocorticoids, it was decreased to 6.9% by the initiation of insulin therapy.

## 3 DISCUSSION

BP is an autoimmune disease that causes bullae, erosions, and erythema on the skin and mucosal surfaces. It is characterized by autoantibodies targeting hemidesmosomal proteins BP180 and BP230 involved in adhesion at the epidermal‐dermal junction.^1^ A wide variety of drugs (diuretics, spironolactone, furosemide, antihypertensives, and antibiotics) have been associated with development of BP in elderly persons.^2^ Recently, there have been increasing reports that use of DPP‐4 inhibitors is also associated with the development of BP.^3^, ^4^, ^5^, ^6^ The rate of DPP‐4 inhibitors use is markedly higher in Japanese people with type 2 diabetes than in Caucasian people,^11^ which may explain why more DPP‐4i‐associated BP cases are reported from Japan.^7^ Several retrospective case‐control studies have compared the frequency of DPP‐4 inhibitor use between BP patients with diabetes and control diabetes patients without BP, demonstrating an association of DPP‐4 inhibitors with the development of BP.^9^, ^10^, ^12^ Accordingly, patients who are using DPP‐4 inhibitors should be warned to report new‐onset diffuse itching, urticarial lesions, or blisters.

We experienced 3 patients with type 2 diabetes who presented BP after treatment with DPP‐4 inhibitors. We confirmed our three cases developed BP as an adverse effect of DPP‐4 inhibitor based on Naranjo scale (an adverse drug reaction probability scale). However, it cannot be denied that amlodipine may be associated with development of BP in Case 1 and 2, because amlodipine is known to be a risk of drug‐induced BP.^2^

Izumi et al reported that DPP‐4 inhibitor‐associated BP featured less prominent erythema clinically and showed little evidence of histological inflammation with scanty infiltration of eosinophils into the skin, suggesting it was an atypical form of noninflammatory BP.^13^ It was also suggested that drug‐induced BP could have a relatively favorable outcome compared with typical BP. Our Cases 1 and 3 were examples of this noninflammatory phenotype of DPP‐4 inhibitor‐associated BP, presenting with less erythema and minor infiltration of eosinophils into the skin on histological examination (Figure 1 and Table 1). On the other hand, there have been reports that most patients with DPP‐4 inhibitor‐associated BP have typical clinical manifestations and histological features of this disease.^10^, ^14^

**Table 1:** Clinical characteristics at the bullous pemphigoid associated with dipeptidyl peptidase‐4 inhibitors in three patients with type 2 diabetes

	Case 1	Case 2	Case 3
Age (yrs)/sex	82/female	89/female	67/male
DPP4‐inhibitors	linagliptin→ teneligliptin	sitagliptin→ vildagliptin	alogliptin
Naranjo scale (points)	3	4	7
Other drugs (/day)	metformin 250 mg		
	miglitol 75 mg		
	amlodipine 5 mg	amlodipine 5 mg	metformin 500 mg
	candesartan 4 mg	candesartan 1 mg	
	loxoprofen 60 mg		
	etizolam 5 mg		
	etizolam 0.5 mg		
Diabetic retinopathy	None	none	none
HbA1c at the onset of BP (%)	7.0	7.9	7.2
eGFR (mL/min/1.73 m^2^)/UAE (mg/gCr)	50.6/–	60.5/–	57.2/3.0
Comorbidity	Hypertension, Cerebral infarction, Intestinal obstruction	Hypertension	Gastric ulcer, Hypertension, Gallstones
Period of DPP‐4 inhibitors administration until onset of BP	16 mo	38 mo	6 mo
anti‐BP180 autoantibody	328 U/mL	3450 U/mL	–
erythema/ blisters	＋/＋	＋/＋	＋/＋
Eosinophil infiltration into the skin	mild	severe	mild
Relapse	＋	–	–
HLA(DQB1*03:01)	–	not examined	＋
Therapy for BP	prednisolone, minocycline, cyclosporine, Steroid pulse therapy, intravenous immunoglobulin therapy	prednisolone, tetracycline, cyclosporine, niceritrol	prednisolone, tetracycline, niceritrol, nicotinamide
Outcomes/Cause	Death: Acute subdural hematoma	Death: Pulmonary metastasis?	Remission

Benzaquen et al reported that discontinuation of DPP‐4 inhibitor therapy had a favorable impact on the outcome of BP in 19 patients with diabetes, because 95% of them achieved clinical remission after stopping DPP‐4 inhibitors and receiving first‐line treatment for BP.^9^ A retrospective case‐control study also demonstrated that clinical outcomes were less favorable among 13 patients with diabetes who continued DPP‐4 inhibitors compared with 19 patients who discontinued DPP‐4 inhibitors, with eight of the 13 patients who continued DPP‐4 inhibitors dying between 2 months and 4.9 years after the initial diagnosis of BP.^10^ These findings suggested that discontinuation of DPP‐4 inhibitor therapy may be associated with better clinical outcomes. Taking the results from these studies together,^9^, ^10^ it can be suggested that administration of DPP‐4 inhibitors should be discontinued immediately when a diagnosis of BP is made. However, we experienced two cases of DPP‐4 inhibitor‐associated BP with unfavorable outcomes despite immediate withdrawal of DPP‐4 inhibitor therapy and initiation of first‐line treatment for BP with an oral steroid and a high‐potency topical steroid. Both patients were elderly women over 80 years old.

In Case 1, skin lesions showed multiple relapses despite discontinuation of DPP‐4 inhibitor therapy and initiation of treatment for BP. She received intravenous steroid pulse therapy, IVIG, and plasmapheresis in hospital on several occasions. Eventually, she died after a fall, probably from cerebral herniation associated with a subdural hematoma. In Case 2, symptoms of BP were improved by treatment with oral prednisolone and cyclosporine, but the patient died after deterioration of her general condition. She may have died of lung metastasis from primary colon cancer, although the possibility that opportunistic pulmonary infection was associated with her death cannot be excluded. It was recently reported that control of BP and relapse of this condition did not differ between patients who stopped or continued treatment with DPP‐4 inhibitors.^15^ This report and our experience suggest that it is important for clinicians to pay close attention to the clinical course of DPP‐4 inhibitor‐associated BP, even after discontinuation of DPP‐4 inhibitor therapy.

The mechanisms responsible for BP associated with DPP‐4 inhibitor therapy remain to be determined. DPP‐4 (CD26) is highly expressed by T cells, especially CD4+ T cells. It is possible that inhibition of DPP‐4 may be associated with development of autoimmune skin diseases, because autoreactive T cells are involved in the pathogenesis of BP.^16^ A previous study demonstrated that the HLA‐DQB1*03:01 allele is a biomarker for genetic susceptibility to BP associated with DPP‐4 inhibitors in a Japanese population,^17^ suggesting an association between HLA class II and this drug‐induced autoimmune disease. Our Case 3 was positive for the HLA‐DQB1*03:01 allele, in agreement with this report (Table 1). We previously demonstrated that sitagliptin, another DPP‐4 inhibitor, reduced circulating CD4 + T cells in patients with type 2 diabetes, especially causing a decline of regulatory T cells.^18^ In fact, a very recent study demonstrated that dysfunction of regulatory T cells is associated with induction of autoantibodies to bullous pemphigoid antigens in mice and humans.^19^ Another possibility is that inhibition of DPP‐4 augments the activity of eotaxin (CCL11), a DPP‐4 substrate, resulting in recruitment of eosinophils to the skin.^20^

In conclusion, we experienced 3 patients with type 2 diabetes who developed DPP‐4 inhibitor‐associated BP. Despite prompt discontinuation of DPP‐4 inhibitor therapy and initiation of first‐line treatment for BP, the outcome was unfavorable in two patients. Accordingly, clinicians should carefully monitor the course of DPP‐4 inhibitor‐associated BP even after withdrawal of DPP‐4 inhibitor therapy, especially in very elderly patients.

## CONFLICT OF INTEREST

None declared.

## AUTHOR CONTRIBUTIONS

YS: treated the patient and drafted the manuscript. TI: wrote the manuscript. EO, SS, and SH: treated the patients. TJ, IU, and KI: reviewed the manuscript. YA: wrote the manuscript. All authors read and approved the final manuscript.
